# Utilizing FUCOM and AHP methods to identify the optimal beekeeping lands: A case study from Mardin, Türkiye

**DOI:** 10.1371/journal.pone.0335784

**Published:** 2025-11-04

**Authors:** Çağrı Mercan, Veysi Acıbuca

**Affiliations:** 1 Department of Mapping and Cadastre, Savur Vocational School, Mardin Artuklu University, Mardin, Türkiye; 2 Department of Horticulture, Kızıltepe Faculty of Agricultural Sciences and Technologies, Mardin Artuklu University, Mardin, Türkiye; Gonbad Kavous University, IRAN, ISLAMIC REPUBLIC OF

## Abstract

Beekeeping plays a vital role in agricultural sustainability and biodiversity conservation, yet identifying ecologically suitable areas for apiculture remains challenging. The objective of this study is to evaluate and compare two Multi-Criteria Decision-Making (MCDM) methods—the Analytical Hierarchy Process (AHP) and the Full Consistency Method (FUCOM)—within a Geographic Information Systems (GIS) framework to identify optimal beekeeping areas in Mardin Province, Türkiye. Nine environmental, climatic, topographical, logistic, and socio-economic factors were selected through literature review, legal regulations, expert consultation, and field observations. Suitability maps were generated and validated using field-verified hive locations and Receiver Operating Characteristic–Area Under the Curve (ROC–AUC) analysis. The results show that land use/cover, proximity to water sources, and precipitation were the most influential factors. Overall, 83% of hive locations coincided with areas classified as moderately suitable or higher. AHP achieved slightly higher predictive accuracy (AUC = 0.774) than FUCOM (AUC = 0.754), while FUCOM required substantially fewer pairwise comparisons, underscoring its efficiency. These findings confirm the robustness of the framework and provide a practical tool for sustainable apicultural land-use planning, offering transferable insights for policymakers, decision-makers, and beekeepers in Türkiye and other regions with similar ecological conditions.

## 1. Introduction

The global population is projected to reach nearly 10 billion by 2050, driving an estimated 59–68% increase in global food demand [1,2]. This unprecedented growth, combined with the compounded effects of climate change, water scarcity, pollution, rapid urbanization, and rising consumption, will exert immense pressure on food security and further weaken ecosystem resilience [3–6]. In this context, developing sustainable strategies capable of securing a stable food supply under dynamic environmental and climatic conditions—while meeting the nutritional needs of a growing population—has become an urgent global priority [7]. Achieving this objective requires the sustainable intensification of both crop and livestock production systems to ensure the well-being of future generations [8].

Pollinators—particularly bees—are indispensable for sustaining agricultural productivity and biodiversity. In addition to producing high-value commodities such as honey, royal jelly, pollen, propolis, and beeswax, beekeeping provides essential ecosystem services that underpin agricultural sustainability and human nutrition [9–12]. Through pollination, bees support the reproduction of a wide variety of wild and cultivated plants [13], thereby enhancing crop yields, preserving floral diversity, and strengthening ecosystem resilience [2,14,15]. These contributions directly and indirectly advance several United Nations Sustainable Development Goals (SDGs), including No Poverty, Zero Hunger, and Life on Land [15].

Nevertheless, widespread declines in bee populations have raised serious concerns for both agriculture and biodiversity [16,17]. The key drivers of this decline include climate change, intensive pesticide use, the spread of parasitic mites such as *Varroa destructor*, viral diseases, and the loss of diverse forage resources [5,18–20]. Consequently, identifying ecologically suitable and sustainable locations for beekeeping has become a pressing research and policy priority.

Beekeeping site selection is inherently complex, shaped by the interplay of biotic and abiotic factors such as climate, vegetation, topography, proximity to water, pesticide exposure, and forage availability [21]. Addressing this complexity requires robust decision-support frameworks that systematically integrate ecological, environmental, and socio-economic parameters. In recent years, the integration of Geographic Information Systems (GIS) and Multi-Criteria Decision-Making (MCDM) methods has emerged as a powerful approach for evaluating agricultural and environmental suitability [12,22–24]. By combining spatial data analysis with structured decision-making, these approaches enable the simultaneous assessment of multiple variables and support evidence-based land-use planning.

Among MCDM techniques, the Analytical Hierarchy Process (AHP) is the most widely applied, offering a hierarchical framework for complex decision problems and enabling expert judgments to be tested for consistency through the Consistency Ratio (CR) [8]. The more recently developed Full Consistency Method (FUCOM), by contrast, reduces the number of pairwise comparisons required while ensuring internal consistency by design [25]. Applying both methods in parallel therefore combines the established reliability of AHP with the efficiency of FUCOM, enabling a more comprehensive evaluation of beekeeping suitability. These approaches were selected because they offer a balance of methodological rigor and practical applicability: AHP is the most established and widely used MCDM method in agricultural and land-use studies, while FUCOM achieves comparable reliability with substantially fewer comparisons [25–27]. Unlike more complex or data-intensive approaches (e.g., fuzzy extensions or hybrid techniques), both methods are transparent, interpretable, and feasible for expert-driven contexts. To our knowledge, this is the first study to apply FUCOM in apicultural land suitability assessment, thereby addressing a key methodological gap in the literature and contributing to decision-support frameworks in pollinator-based agriculture.

Considering the critical importance of pollinator-based agriculture, the multidimensional nature of site suitability assessment, and the analytical potential of MCDM approaches, this study pursues three primary objectives:

(i) to identify suitable areas for beekeeping through a dual-method GIS–MCDM framework,
(ii) to compare the performance of the FUCOM with the widely applied AHP, and
(iii) to validate the resulting suitability maps using field-based hive location data.

The outcomes aim to provide decision-makers with a reliable methodological framework for sustainable beekeeping land-use planning, offer practical guidance for local honey producers, and contribute to the achievement of multiple SDGs.

## 2. Literature review

The integration of GIS with MCDM techniques has become increasingly prominent in assessing suitable areas for beekeeping [28,29]. This combined framework enables a systematic, spatially explicit, and quantitatively robust evaluation of ecological, environmental, and socio-economic factors that influence apicultural productivity. Unlike purely qualitative assessments, GIS–MCDM approaches allow multiple variables—such as climate, floral resources, and management practices—to be considered simultaneously, thereby enhancing both the accuracy and transparency of suitability analyses [8,12].

A wide range of studies has demonstrated the versatility of GIS–MCDM approaches across diverse contexts. In Türkiye, large-scale modeling based on meteorological and environmental data identified 33 provinces suitable for migratory beekeeping [9]. In Greece, Marnasidis [30] applied GIS–MCDM to map areas conducive to beekeeping development, while Cotrina-Sanchez [31] employed AHP-weighted biophysical and socio-economic criteria to support rural communities in Peru. Similarly, Sarvia [32] modeled the spatial distribution of hives in Italy, Kamga [2] developed a fuzzy inference system for Quebec (Canada), and Tennakoon [33] applied fuzzy AHP in Queensland (Australia). Collectively, these studies highlight the growing reliance on GIS–MCDM frameworks—particularly the dominance of AHP—for identifying suitable beekeeping areas [8,12,28,29,31,34–43].

Recent contributions have expanded the methodological scope of beekeeping suitability research. For example, Astuti [44] assessed Artificial Intelligence (AI) applications for apiculture under climate change, while Gomez-Fernandez [45] and Zapata-Hernández [20] analyzed global evidence on climate-driven risks to bee populations. Other studies have emphasized the use of open-source spatial data (e.g., OpenStreetMap) [46] or the ecological importance of forested landscapes in supporting floral resources and pollinator health [47].

Among MCDM techniques, AHP remains the most widely applied in beekeeping suitability studies [8,12,28,29,31,34–41]. However, a growing body of literature has adopted alternative methods such as the Technique for Order of Preference by Similarity to Ideal Solution (TOPSIS), VIseKriterijumska Optimizacija I Kompromisno Resenje (VIKOR) [48,49], Preference Ranking Organization Method for Enrichment Evaluation (PROMETHEE) [43], Logarithmic Methodology of Additive Weights (LMAW) [50], Method Based on the Removal Effects of Criteria (MEREC) [51], Simultaneous Evaluation of Criteria and Alternatives (SECA) [52], Stepwise Weight Assessment Ratio Analysis II (SWARA II) [53], the Best–Worst Method (BWM) [54], and the Objective Pairwise Adjusted Ratio Analysis (OPARA) [55], as well as various fuzzy-based approaches [2,33]. Despite their proven effectiveness in other domains, only a few of these methods, such as TOPSIS and VIKOR, have been applied to beekeeping suitability [42,43], while many others have yet to be sufficiently explored in Mardin or comparable ecological contexts.

FUCOM, introduced by Pamučar [27], is an MCDM approach designed to derive reliable weights with a minimal number of pairwise comparisons. By requiring fewer evaluations than traditional methods and ensuring full internal consistency, FUCOM offers an efficient and mathematically robust alternative, particularly in contexts where expert availability is limited [56]. FUCOM has been successfully applied in numerous domains, including hazardous material transport, air traffic route planning, sustainable supplier selection, bridge site assessment, wind farm location planning, landfill site selection, groundwater evaluation, mining potential mapping, and agricultural land suitability [25,57–65]. However, FUCOM’s flat ranking structure constrains its capacity to model multi-layered decision problems, and the absence of fuzzy or probabilistic extensions in its classical form limits its ability to address uncertainty. Although fuzzy FUCOM variants have been proposed [66], the present study applies the classical FUCOM method for methodological simplicity and consistency with the crisp consensus data obtained from experts.

While AHP has long dominated apicultural suitability research, FUCOM has never been systematically applied in this domain. By testing FUCOM alongside AHP in Mardin Province, this study fills a methodological gap and offers novel evidence on the applicability of FUCOM to apicultural land suitability assessment.

## 3. Materials and methods

**Fig 1:**
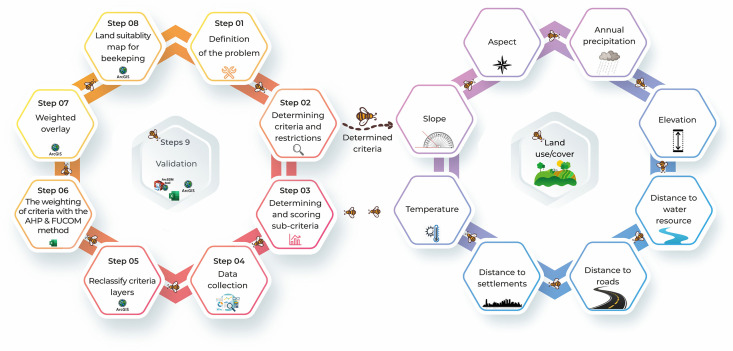
Conceptual methodological process. All diagrams were produced by the authors. No copyrighted or proprietary data were used.

The methodological design of this study followed a structured workflow consisting of sequential steps, beginning with problem definition and progressing through the generation and validation of suitability maps. These interrelated stages established a systematic foundation for data processing, reclassification, weighting, and overlay analysis. An overview of the methodological framework is presented in Fig 1, which not only summarizes the workflow but also outlines the environmental, climatic, and accessibility criteria applied in the analysis.

### 3.1 Study area

**Fig 2:**
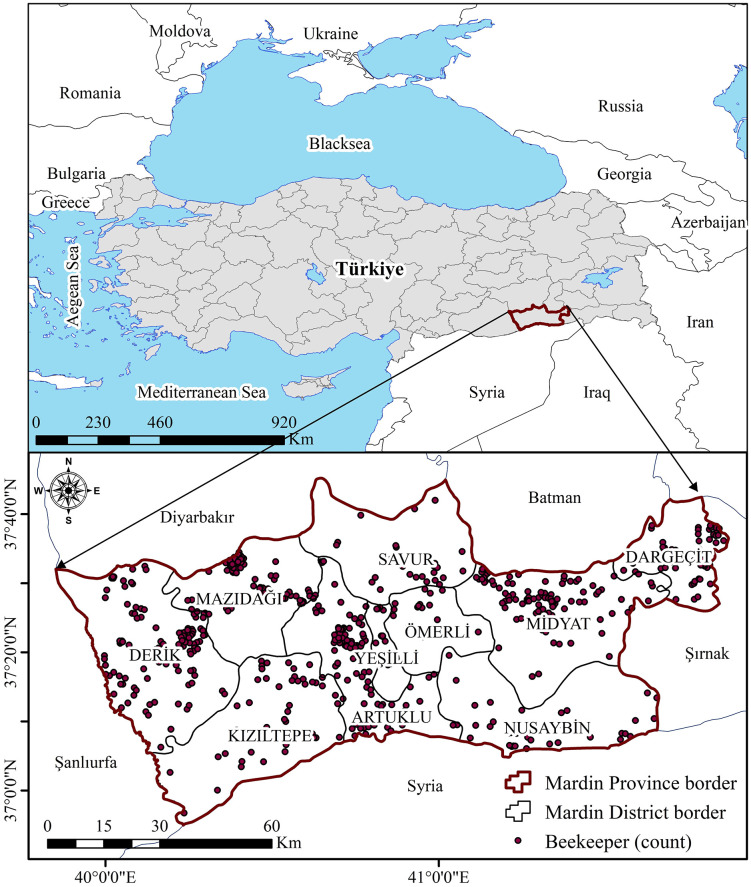
Location of the study area. Country boundaries were obtained from Natural Earth (public domain). Administrative boundaries were derived from the General Directorate of Mapping, Türkiye (open-access vector data, no copyright restrictions). Beekeeper location data were collected by the authors during field surveys using GPS devices. All maps were created by the authors using publicly available datasets processed in ArcGIS without any proprietary basemap. No copyrighted material was used.

Mardin, located in Türkiye’s Southeastern Anatolia Region, covers an area of approximately 8,781 km². Geographically, it lies between 39°56′–42°54′ E and 36°55′–38°51′ N (Fig 2). Administratively, Mardin consists of ten districts and is characterized by both Mediterranean and continental climatic features. Summers are typically hot and dry, while winters are cold and rainy [67,68]. Long-term meteorological records indicate an average annual temperature of 16.2 °C, a mean daily sunshine duration of 8.1 hours, and annual precipitation of 673.5 mm [69].

Beekeeping in the region is primarily practiced during spring, when floral resources are abundant, as hot and dry summers restrict nectar availability. The flowering season of major melliferous plant species generally extends from March to June [70]. Topographically, the province comprises fertile lowland plains in the south, dominated by intensive agriculture, and mountainous northern areas, which host diverse endemic flora and provide favorable conditions for apiculture [68]. Mardin was selected as the study area due to ongoing state-supported apiculture initiatives, the absence of prior spatially explicit assessments of beekeeping suitability, and the need for scientific insights to guide sustainable beekeeping practices.

### 3.2 Criteria selection process and ethical considerations

The selection of evaluation criteria followed a multi-stage process that combined a systematic review of the relevant literature [8,9,12,21,28,29,34–40,42,43,71–74], an assessment of national legal regulations, consultations with regional experts, and direct field observations. This integrative approach ensured that the criteria were scientifically justified, contextually relevant, and legally compliant. Notably, Turkish legislation prohibits the placement of beehives within certain distances of highways and settlement centers, and these regulatory constraints were explicitly incorporated into the decision framework to enhance the practical applicability of the study [8]. To maintain a balanced perspective, the selected criteria were grouped into three overarching categories: environmental and climatic factors (land use/land cover, distance to water sources, precipitation, temperature), topographical factors (slope, aspect, elevation), and logistic and socio-economic factors (distance to settlements, distance to roads).

Expert consultation was carried out using a snowball sampling strategy, during which approximately 615 beehives across the province were located and recorded with GPS. Valuable support from the Mardin Provincial Directorate of Agriculture and Forestry facilitated access to beekeepers during this process. From this broader pool, a final group of 20 professional beekeepers, 2 biologists, and 3 agricultural engineers—each with direct knowledge of apiculture and regional conditions—volunteered to serve as expert participants. Prior to the panel sessions, all participants were briefed on the research objectives and the methodological framework, including FUCOM and AHP. Findings from the literature review and regulatory analyses were then presented, after which the experts collectively identified and refined the most relevant criteria for assessing apicultural suitability in the study area. Only those criteria that achieved full consensus were retained for further analysis. The final set of criteria, along with their justifications and supporting references from the literature, are provided in Table 1. This tabular summary ensures transparency regarding the rationale behind each criterion and allows readers to trace the alignment of our choices with prior research.

**Table 1:** Evaluation criteria, justifications, and supporting references for beekeeping suitability.

Criteria	Rationale for Selection	References
Land use/ Land cover	Represents nectar and pollen sources. Unsuitable areas (urban, wetlands, water bodies, etc.) were restricted.	[8,28,29,34–37,39,40,42,71,72]
Distance to water sources	Essential for hive cooling, honey production, and maintaining vegetation diversity.	[8,28,29,34,35,37,39,40,42]
Annual precipitation	Determines vegetation growth; drought is a major limitation in Mardin.	[8,28,29,34,36,39,42]
Slope	Affects accessibility and hive management.	[8,12,28,29,42]
Aspect	Orientation affects microclimate and honey yield.	[8,28,29,42,73]
Elevation	Influences climate and flora. < 550 m restricted due to agricultural pesticide use.	[8,28,29,34,35,39,42]
Temperature	Bees are highly sensitive to temperature; March–June averages used.	[8,21,36,71,74]
Distance to settlements	Avoids human–bee conflicts; unsuitable within 200 m of settlements.	[9,28,29,39,40,42]
Distance to roads	Roads are important for hive transport, but unsuitable within 200 m due to pollution and legislation.	[8,9,28,29,34–36,39,40 42]

Following the determination of criteria, the weighting methods were introduced to the experts in simplified form. Instead of collecting and publishing raw scoring tables from each individual expert, a consensus-based approach was adopted: participants jointly discussed the relevance and importance of each criterion until agreement was reached. This consensus was then used to assign criterion weights within the FUCOM and AHP frameworks. The subsequent calculations and spatial analyses were conducted by the authors using ArcGIS (v.10.8), which produced preliminary suitability maps. These outputs were later presented back to the expert group in a feedback session, during which the validity and applicability of the results were confirmed.

From an ethical standpoint, the study did not involve biological sampling, experimental interventions on humans or animals, or research in restricted areas requiring formal ethical approval. All data collection relied solely on professional expertise and publicly available datasets. All participants were adults and provided informed verbal consent prior to their involvement. This consent was obtained in a group setting, in the presence of the researchers and all participants, and was verbally confirmed by each individual before proceeding. The group-based setting functioned as a mechanism of mutual witnessing and documentation, thereby ensuring transparency and accountability. No personal or sensitive data were recorded, and participation posed no risk to the individuals involved. Because the study did not include human or animal experimentation, biological sampling, surveys or questionnaires, or personal data, approval from an Institutional Review Board (IRB) or ethics committee was not required.

### 3.3 Data collection

The datasets used in this study were obtained exclusively from openly accessible and widely recognized sources that are free of charge and commonly employed in scientific research. This approach ensured both transparency and reproducibility, while avoiding any copyright or licensing concerns.

The provincial and district boundary data for Mardin were provided by the General Directorate of Mapping (https://www.harita.gov.tr/urun/turkiye-mulki-idare-sinirlari/232, accessed on 02/01/2025). Land use/cover information was derived from the CORINE Land Cover database (2018 edition) at a 1:100,000 scale in vector format. Distances to water sources, roads, and settlements were calculated using data from OpenStreetMap (https://www.openstreetmap.org/, accessed on 03/01/2025), with Euclidean distance functions applied for all calculations.

Elevation data were obtained from the Shuttle Radar Topography Mission (SRTM), provided by NASA (https://earthexplorer.usgs.gov/, accessed on 03/01/2025). This dataset, available in raster format with a spatial resolution of 28 m, was further processed to derive slope and aspect layers.

Climatic variables, including temperature and precipitation, were sourced from the WorldClim database (http://www.worldclim.org/, accessed on 02/01/2025) in raster format, with a spatial resolution of 30 arc-seconds (~1 km²).

All datasets were standardized and integrated within a GIS environment after being transformed into the WGS 84 – Universal Transverse Mercator (UTM) Zone 37 coordinate system.

### 3.4 Reclassification of criteria and suitability thresholds

Following the selection of criteria, all sub-criteria were standardized and reclassified into five suitability levels: very high (5), high (4), moderate (3), low (2), and very low (1). Suitability thresholds were defined through a combination of literature review and expert consultation. Areas deemed unsuitable due to legal, social, or technical constraints were classified as restricted (0) and excluded from further analysis (Table 2).

**Table 2:** Reclassification values and hierarchical scores for the sub-criteria.

Criteria ↓	Score →	0 (Restricted)	1 (Very low suitable)	2 (Low suitable)	3 (Moderate suitable)	4 (High suitable)	5 (Very high suitable)
Land Use/Cover (Corine Code)		111, 112, 121, 124, 131, 133, 142, 331, 411, 511, 512	211, 212, 242, 332, 334		221, 222, 243, 333	311, 312, 313, 324	231, 321
Dist. to water resource (m)			>4000	3000-4000	2000-3000	1000-2000	0-1000
Annual Precipitation (mm)			354-470	470-559	559-640	640-701	701-838
Slope (%)			>60	45-60	30-45	15-30	0-15
Aspect			N	NW, NE	W, E	SE, SW	F, S
Elevation (m)		348-550	>1150	1000-1150	850-1000	700-850	550-700
Temperature (°C)			15-17	17-18	18-19	19-20	20-21
Dist. to settlements (m)		0-200	200-500	500-1500	1500-2000	2000-2500	>2500
Dist. to roads (m)		0-200	>4000	3000-4000	2000-3000	1000-2000	200-1000

**Fig 3:**
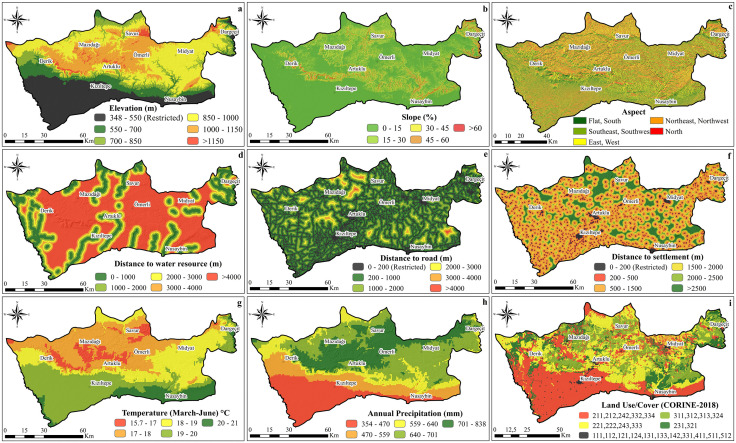
Thematic maps for the assessed criteria; (a) Elevation, (b) Slope, (c) Aspect, (d) Distance to water resource, (e) Distance to road, (f) Distance to settlement, (g) Temperature (March-June), (h) Annual precipitation, (i) Land use/cover. All maps were created by the authors using ArcGIS software. Administrative boundaries were obtained from the General Directorate of Mapping, Türkiye (open-access vector data, no copyright restrictions). Thematic layers were generated from publicly available datasets described in the Data Collection section, including CORINE Land Cover (EEA, 2018), SRTM (NASA), WorldClim, and OpenStreetMap (vector data). No copyrighted or proprietary data were used.

This reclassification formed the basis for generating thematic suitability maps. In these maps, shades of green indicate higher suitability, shades of red represent lower suitability, and restricted areas are displayed in black (Fig 3).

### 3.5 Weighting of criteria

Criteria weighting was performed using FUCOM and AHP, following the consensus-based procedure outlined in Section 3.2. This approach ensured that the derived weights reflected both expert knowledge and practical experience, while also enabling a comparative evaluation of the two methods.

#### 3.5.1 FUCOM.

FUCOM method involves three essential steps: (i) establishing the rank order of the criteria, (ii) defining comparative priorities between adjacent criteria, and (iii) calculating the final weights through a linear programming model.

The method imposes two key consistency conditions: (1) the ratios of the derived weights must correspond to the experts’ stated comparative priorities, and (2) the transitivity property must be satisfied across all criteria. To assess compliance with these conditions, FUCOM calculates the deviation from full consistency (DFC), where a lower DFC value indicates higher internal consistency in the derived weights.

Through this process, FUCOM generates weight coefficients that are both mathematically validated and derived with substantially fewer pairwise comparisons compared to classical approaches. The procedure was applied in accordance with the original formulation by Pamučar [27]. The method comprises three main stages, each accompanied by its respective mathematical formulation, as outlined below.

**Step 1.** The evaluation criteria are prioritized according to their level of significance. In Equation (1), the decision criteria are ranked from the most to the least important. The variable *k* denotes the level of importance of the criteria. If two criteria are considered equally important, the “=” sign is used instead of “>”:


$$C_{j(1)}>C_{j(2)}>\cdots >C_{j(k)}\mathrm{\backslash ]}$$ (1)

**Step 2.** The decision maker’s preferences are then used to establish the relative significance of adjacent criteria. This yields a vector of comparative priorities for the evaluation criteria. The expression φ_k/(k+1)_ represents the relative importance of criterion C_j(k)_ compared to criterion C_j(k+1)_. Accordingly, the vector of comparative priorities is expressed as:


$$\Phi =(\varphi_{1/2},\varphi_{2/3},\varphi_{3/4},\cdots ,\varphi_{k/(k+1)})$$ (2)

**Step 3.** The weight values for the criteria are then determined subject to two conditions:

- **Condition 1.** The ratio of the weight coefficients must equal the priority values derived in Step 2:

$$\frac{\omega_{k}}{\omega_{k+1}}=\varphi_{k/(k+1)}$$ (3)

**Condition 2.** The weight coefficients at the final should ensure mathematical transitivity; that is

$\varphi_{\frac{k}{\left(k+1\right)}}⊗\;\varphi_{\frac{\left(k+1\right)}{\left(k+2\right)}}=\;\varphi_{\frac{k}{\left(k+2\right)}}\;$. Since $\varphi_{\frac{k}{\left(k+1\right)}}=\;\frac{\omega_{k}}{\omega_{k+1}}$ and $\varphi_{\frac{\left(k+1\right)}{\left(k+2\right)}}=\;\frac{\omega_{k+1}}{\omega_{k+2}}\;$, that $\frac{\omega_{k}}{\omega_{k+1}}\;⨂\;\frac{\omega_{k+1}}{\omega_{k+2}}=\frac{\omega_{k}}{\omega_{k+2}}\;$ is obtained. Another requirement that needs to be met to obtain the final values of the weight coefficients of the criteria is illustrated in Equation (4)


$$\frac{\omega_{k}}{\omega_{k+2}}=\varphi_{k/(k+1)}\;⨂{\;\varphi }_{\left(k+1)/(k+2\right)}\;$$ (4)

When both conditions are satisfied, the requirement for maximum consistency is achieved, with the deviation from full consistency (DFC) equal to zero (χ = 0).

To determine the final weight coefficients, the following linear programming model is solved:


Min χ

subject to:


$$\left|\frac{\omega_{j(k)}}{\omega_{j(k+1)}}-\;\varphi_{k/(k+1)}\right|\leq \chi \;,\;\forall j$$

$$\left|\frac{\omega_{j(k)}}{\omega_{j(k+2)}}-\varphi_{k/(k+1)}⨂\varphi_{\left(k+1)/(k+2\right)}\right|\leq \chi \;,\;\forall j$$ (5)

$$\sum_{j=1}^{n}\omega_{j}=1,\;\omega_{j}\geq 0\;,\;\forall j$$

By solving this model, the final weights of the evaluation criteria ($\omega_{\text{1}}$, $\omega_{\text{2}}$, …, $\omega_{n}$)^*T*^ and the DFC value (χ) are obtained. Table 3 summarizes the rankings and pair-wise comparisons derived from FUCOM method for this study

**Table 3:** Ranking and comparison values of the criteria for FUCOM.

Criteria	Rank	Comparisons
Land Use/Cover	1	1
Dist. to water resource	2	3
Precipitation	3	4
Slope	4	5
Aspect	5	6
Elevation	6	7
Temperature	7	8
Dist. to settlements	8	9
Distance to roads	9	9

Deviation from full consistency χ = 0.00.

#### 3.5.2 AHP.

AHP, developed by Saaty in 1977, is one of the most widely applied MCDM methods, offering an analytical approach to structuring and solving complex decision problems [75,76]. It has been applied extensively across various fields, including agriculture and environmental management, due to its ability to integrate both qualitative judgments and quantitative analysis. AHP structures a decision problem into a hierarchical model, with the overall goal at the top, criteria and sub-criteria at intermediate levels, and the alternatives at the bottom. Decision-making proceeds through pairwise comparisons of criteria and alternatives, followed by a consistency check to ensure the reliability of judgments.

The AHP method was applied in this study according to the original formulation of Saaty [75,76] and consists of six main stages:

**Step 1. Pairwise comparisons.** Criteria and alternatives are evaluated through pairwise comparisons based on expert opinion and literature review (Equation 6). A numerical scale ranging from 1 (equal importance) to 9 (extreme importance) is used, as proposed by Saaty [75].


$$A=\left[\begin{array}{ccc} a_{11} & a_{12} & \ldots \\ a_{21} & a_{22} & \cdots \\ \vdots & \vdots & \ddots \\ a_{i1} & a_{i2} & \cdots \end{array}\;\begin{array}{c} a_{1j}\\ a_{2j}\\ \vdots \\ a_{ij} \end{array}\right]$$ (6)

Here, a_ij_ represents the pairwise comparison between criterion *i* and criterion *j*.

**Step 2. Normalization.** Each element of the matrix is normalized by dividing it by the sum of its respective column (Equation 7). As a result, the sum of each column in the normalized matrix equals 1:


$$b_{ij}=\frac{a_{ij}}{\sum_{i=1}^{n}a_{ij}},\;i,\;j=1,\;2,\;\ldots ,n$$ (7)

**Step 3. Priority vector.** The importance weights of each criterion are calculated by summing the elements of each row of the normalized matrix, dividing by the matrix size, and taking the average. These values form the priority vector (Equation 8):


$$W_{\dot{\text{I}}}=\frac{\sum_{j=1}^{n}b_{ij}}{n},\;i=1,\;2,\;\ldots ,\;n$$ (8)

**Step 4. Weighted sum vector.** The comparison matrix *A* is multiplied by the priority vector *W* to obtain the weighted sum vector *C* (Equation 9):


C=A×W (9)

**Step 5. Consistency Index (CI).** To evaluate the consistency of judgments, the Consistency Index (CI) is calculated using the maximum eigenvalue (λ_max_) and the number of criteria *n* (Equation 10):


$$CI=\frac{\lambda max-n}{n-1}$$ (10)

To obtain *λ*_max_, each element of the weighted sum vector *C* is divided by the corresponding element of the priority vector *W*, and the average of these values is taken.

**Step 6. Consistency Ratio (CR).** The Random Index (RI), which depends on the size of the comparison matrix, is obtained from established tables in the literature [75]. The Consistency Ratio (CR) is then calculated (Equation 11). If CR < 0.10, the matrix is considered consistent:


$$CR=\frac{CI}{RI}$$ (11)

Table 4 displays the pairwise comparison matrix along with the CI, λ_max_, and CR values. Table 5 presents the weight values obtained using the FUCOM and AHP methods.

**Table 4:** Pairwise comparison matrix for AHP.

Pairwise comparison matrix	C1	C2	C3	C4	C5	C6	C7	C8	C9
Land Use/Cover (C1)	1	4	5	6	7	7	8	9	9
Dist.water resource (C2)	1/4	1	2	2	3	4	6	6	8
Annual Precipitation (C3)	1/5	1/2	1	2	3	4	4	5	7
Slope (C4)	1/6	1/2	1/2	1	2	3	4	5	6
Aspect (C5)	1/7	1/3	1/3	1/2	1	2	3	4	5
Elevation (C6)	1/7	1/4	1/4	1/3	1/2	1	2	3	5
Temperature (C7)	1/8	1/6	1/4	1/4	1/3	1/2	1	2	3
Dist. Settlements (C8)	1/9	1/6	1/5	1/5	1/4	1/3	1/2	1	2
Dist. Roads (C9)	1/9	1/8	1/7	1/6	1/5	1/5	1/3	1/2	1

Scale: 1-Equal importance, 3-Moderate importance, 5-Strong importance, 7-Very strong importance, 9-Extreme importance, 2,4,6 and 8-Intermediate importance [76].

Max. eigenvalue (λ_max_) = 9.562, n = 9, Consistency index (CI) = (λ_max_-n)/(n-1) = 0.070, Random index (RI) = 1.45, Consistency ratio (CR) = (CI/RI) = 0.048.

**Table 5:** Comparisons of weighted values based on AHP and FUCOM.

Criteria	AHP	FUCOM	Mean	Std. dev.	CV
Land Use/Cover	0.401	0.410	0.405	0.005	1.13
Dist. to water resource	0.171	0.137	0.154	0.017	11.04
Precipitation	0.134	0.102	0.118	0.016	13.41
Slope	0.101	0.082	0.091	0.009	10.38
Aspect	0.069	0.068	0.069	0.000	0.59
Elevation	0.050	0.059	0.054	0.004	8.00
Temperature	0.033	0.051	0.042	0.009	21.17
Dist. to settlements	0.024	0.046	0.035	0.011	31.02
Distance to roads	0.017	0.046	0.031	0.014	44.77

Std. dev.: Standard deviation, CV: Coefficient of variation.

### 3.6 Generation of the beekeeping suitability map

**Fig 4:**
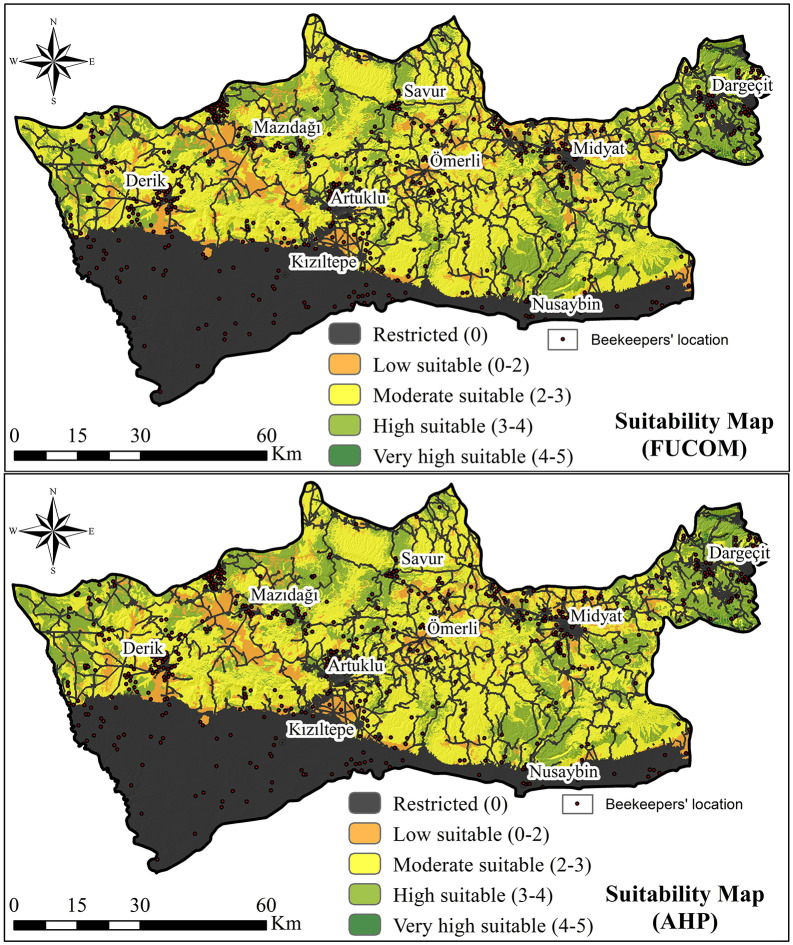
Land suitability maps for beekeeping. Administrative boundaries were obtained from the General Directorate of Mapping, Türkiye (open-access vector data, no copyright restrictions). The suitability map was generated by overlaying weighted thematic layers produced by the authors according to the FUCOM–AHP model. Beekeeper location points were collected by the authors during field surveys using GPS devices. All map elements were created by the authors; no copyrighted or proprietary data were used.


The standardized criterion layers were combined using the Weighted Overlay tool in ArcGIS. Separate overlays were produced with FUCOM- and AHP-derived weights to enable method comparison. The pixel-level beekeeping suitability index (ASI) was computed as a weighted linear combination (Eq. 12). The resulting maps were classified into five suitability levels: very low (1), low (2), moderate (3), high (4), and very high (5), while restricted areas were masked and assigned a value of 0 (Fig 4).

$${ASI}_{i}=\sum_{j=1}^{n}W_{j}X_{ij}$$ (12)

where n is the number of criteria, W_j_ is the normalized weight of criterion j, and X_ij_ is the standardized score of pixel i for criterion j [77,78].

### 3.7 Validation of results

To ensure the methodological reliability and robustness of the beekeeping suitability assessment, the results derived from FUCOM and AHP frameworks were validated through a set of complementary approaches. These approaches were designed to evaluate both spatial accuracy and methodological consistency, thereby strengthening the credibility of the final suitability maps.

#### 3.7.1 Field-based validation with hive locations.

**Fig 5:**
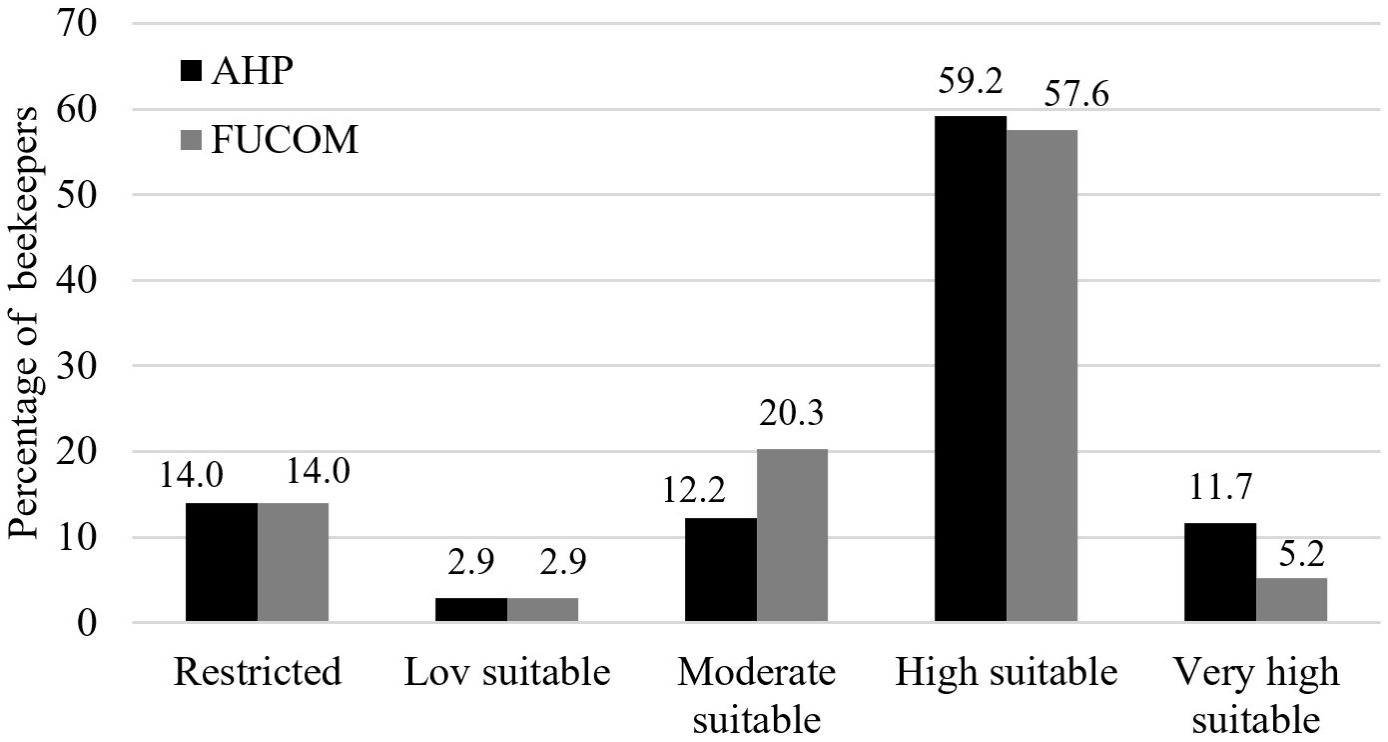
Comparison of suitability class and existing beekeepers. This figure compares classified suitability areas with actual beekeeping locations to evaluate spatial consistency.

A total of 615 active hive locations were recorded across the study area using handheld GPS devices. These points were overlaid on the suitability maps, and pixel values corresponding to each location were extracted in ArcGIS. Hive points were then classified into suitability categories, enabling the calculation of the proportion of hives in each class (Fig 5). Photographs of selected hives recorded during the fieldwork are shown in S1 Fig. The degree of overlap between the field-recorded hive locations and the predicted suitability zones provided a direct measure of the model’s spatial accuracy. The high correspondence between hive locations and the “moderately suitable,” “highly suitable,” and “very highly suitable” areas was considered strong evidence of model validity.

#### 3.7.2 Statistical validation using ROC–AUC.

Receiver Operating Characteristic (ROC) analysis and the Area Under the Curve (AUC) were applied to quantitatively evaluate predictive performance. The ROC–AUC procedure was implemented in ArcSDM for ArcGIS, using suitability rasters as input and field-recorded hive locations as the presence dataset. Absence data were not included. AUC values above 0.5 were considered acceptable indicators of predictive accuracy.

#### 3.7.3 Expert feedback and cross-validation.

Preliminary suitability maps were presented to professional beekeepers, biologists, and agricultural engineers for further evaluation. Experts confirmed that the spatial distribution of high-suitability zones aligned with their field experience and knowledge of regional apicultural practices.

#### 3.7.4 Consistency and reliability of weighting methods.

The Consistency Ratio (CR) in AHP and the Deviation from Full Consistency (DFC) in FUCOM were used to assess methodological soundness. Both indicators confirmed that the derived weights were logically coherent and internally consistent.

## 4. Result

### 4.1 Evaluation of criteria outputs

Nine criteria influencing beekeeping suitability in the study area were mapped after reclassification (Fig 3 and Table 1). Pasture and natural grasslands emerged as the most critical land-use categories, covering approximately 116,551 ha, while forest and shrublands accounted for nearly 163,573 ha. These areas are ecologically important for apiculture, as previous research emphasizes the role of forested landscapes in protecting bees from wind and rain and in providing diverse floral resources [71].

Water availability was unevenly distributed, with districts such as Dargeçit having the greatest access to water sources, while others remained more limited. Precipitation and elevation patterns also revealed strong north–south contrasts: upland districts such as Ömerli and Savur received substantially more rainfall (>690 mm) compared to lowland areas like Kızıltepe (<460 mm). Similarly, slope values were steepest in Dargeçit (>23%), whereas Kızıltepe remained predominantly flat (<6%).

Temperature conditions during the flowering season varied from ~17 °C in upland districts such as Mazıdağı to nearly 20 °C in lowland districts such as Nusaybin and Kızıltepe. Settlement and road densities showed notable contrasts: Kızıltepe had the highest density of both, reflecting intensive agricultural activity and associated pesticide risks, whereas Savur, with the lowest density, remained the least disturbed.

Collectively, these results highlight the ecological heterogeneity of Mardin Province. Upland districts generally offered favorable climatic and topographic conditions for apiculture, whereas lowland districts were more limited by reduced rainfall, higher temperatures, and intensive agricultural practices.

### 4.2 AHP and FUCOM

The deviation from full consistency (DFC) in the FUCOM method was calculated as χ = 0.00 (Table 3). For AHP, the Consistency Ratio (CR) was 0.049 (Table 4), well below the commonly accepted threshold of 0.10. These values confirm that both methods generated internally consistent weights.

The relative importance of the evaluation criteria was determined using both AHP and FUCOM (Table 5). Land use/cover emerged as the most influential factor, followed by distance to water sources, precipitation, slope, aspect, elevation, temperature, distance to settlements, and distance to roads. According to AHP, land use/cover accounted for 40.1% of the total weight, compared to 41.0% under FUCOM.

### 4.3 Land suitability maps

Beekeeping suitability maps were generated using weights derived from both FUCOM and AHP (Fig 4). The spatial distribution of suitability classes by district is summarized in S1 Table and S2 Table.

Restricted areas were most prominent in Kızıltepe, where intensive agricultural practices and pesticide use limit apicultural potential; these zones accounted for about 41% of the province in both methods. Lands classified as low suitability were concentrated in Mazıdağı, reflecting limited access to water sources. Moderately suitable areas dominated Savur, while highly suitable areas were clustered in Midyat and Dargeçit. Very highly suitable zones were relatively scarce but mainly observed in Dargeçit.

Overall, more than half of Mardin Province fell into the moderately, highly, or very highly suitable categories. The close agreement between FUCOM and AHP outputs—with only minor differences in class proportions—demonstrates the robustness of the dual-method approach. According to both methods, the highest suitability was observed in upland districts such as Midyat, Savur, and Dargeçit, whereas Kızıltepe and Yeşilli consistently ranked lowest. Full district-level distributions are provided in S1 and S2 Tables.

### 4.4 Validation results

Validation results consistently confirmed the robustness of the proposed framework.

**Fig 6:**
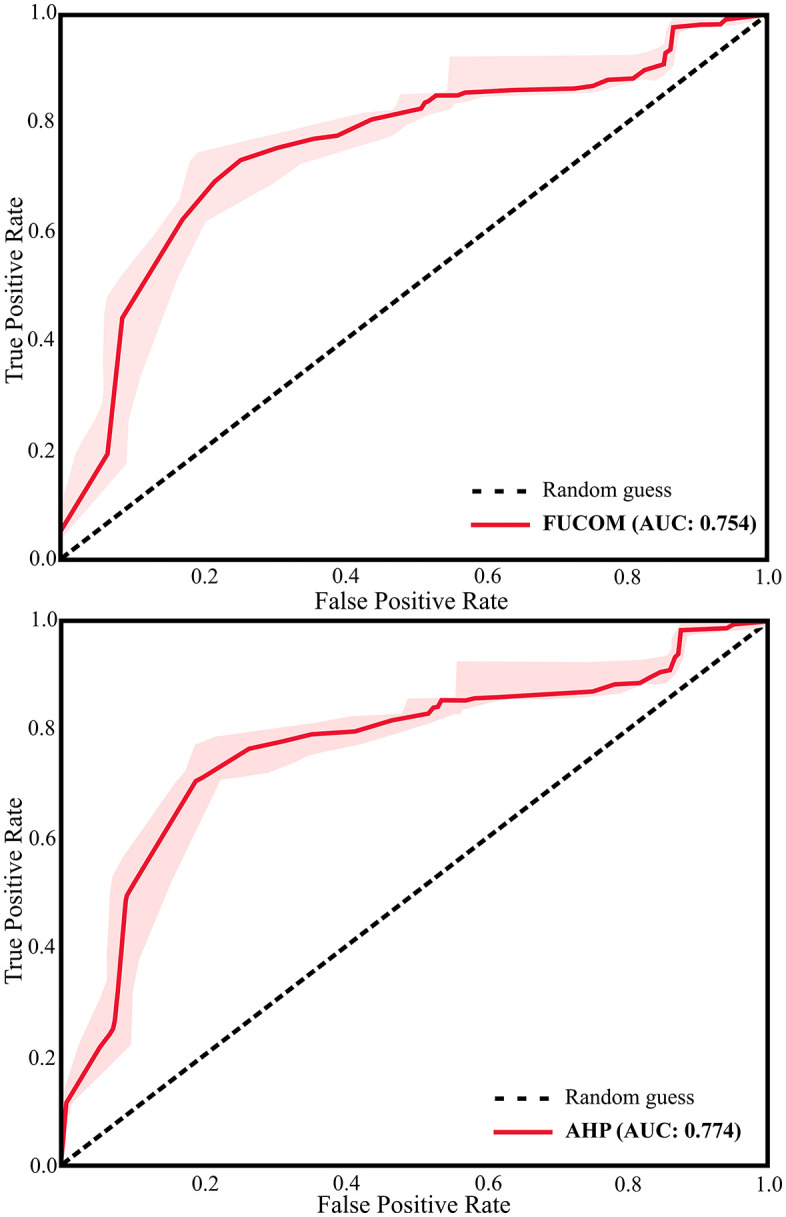
The area under the ROC curve (AUC) for beekeeping. This figure presents the ROC curve showing model accuracy and predictive performance.

- **Field-based validation:** Of the 615 recorded hive locations, 83% were located within areas classified as moderately, highly, or very highly suitable (Fig 5). This high degree of overlap provided strong evidence of the spatial accuracy of the suitability maps.
- **Statistical validation:** ROC–AUC analysis yielded values of 0.774 for AHP and 0.754 for FUCOM (Fig 6). Both exceeded the 0.5 threshold for acceptable performance, indicating good predictive accuracy. The slightly higher AUC for AHP suggests that its hierarchical structure and consistency checks offered a modest advantage over FUCOM.
- **Consistency checks:** The Consistency Ratio (CR) for AHP and the Deviation from Full Consistency (DFC) for FUCOM both confirmed the logical coherence and mathematical reliability of the expert judgments.
- **Expert feedback:** The spatial analysis of suitable zones was corroborated by an expert panel of beekeepers, biologists, and agricultural engineers, who affirmed the high degree of congruence between the model’s predictions and real-world conditions.

Taken together, the convergence of empirical field evidence, statistical metrics, and expert evaluation underscores the reliability and practical applicability of the proposed methodological framework for apicultural land-use planning.

## 5. Discussion

### 5.1 Evaluation of criteria

The selection of evaluation criteria is critical for ensuring the reliability and applicability of beekeeping suitability assessments. The nine factors considered in this study—land use/cover, elevation, slope, aspect, temperature, precipitation, and distance to water sources, roads, and settlements (Table 1)—are consistent with those widely used in previous research. For example, studies in Türkiye [8,12,29,42,43], Australia [79], and Canada [2] have employed similar combinations of climatic, topographic, and accessibility-related variables, underscoring their relevance across diverse ecological contexts.

Although the relative weights of these criteria vary depending on regional conditions, land use/cover consistently emerges as the dominant factor. Previous studies reported weights ranging from 31.7% [8] to 46.4% [12], which closely align with the present findings, where land use/cover was again the most influential determinant. This consistency highlights the central role of land use/cover in capturing both ecological suitability and anthropogenic pressures relevant to apiculture.

At the same time, the applicability of these criteria is inherently context-specific. The ecological and socio-economic conditions of Mardin shaped their selection and weighting, which means their transfer to other regions should be undertaken cautiously. Local differences in vegetation, climate, and land-use dynamics may require adaptation or recalibration to ensure accurate suitability assessments.

### 5.2 Advantages and disadvantages of FUCOM and AHP approaches

MCDM methods play a central role in generating reliable suitability maps. In this study, the AHP and FUCOM were compared to assess their relative strengths and limitations.

**Fig 7:**
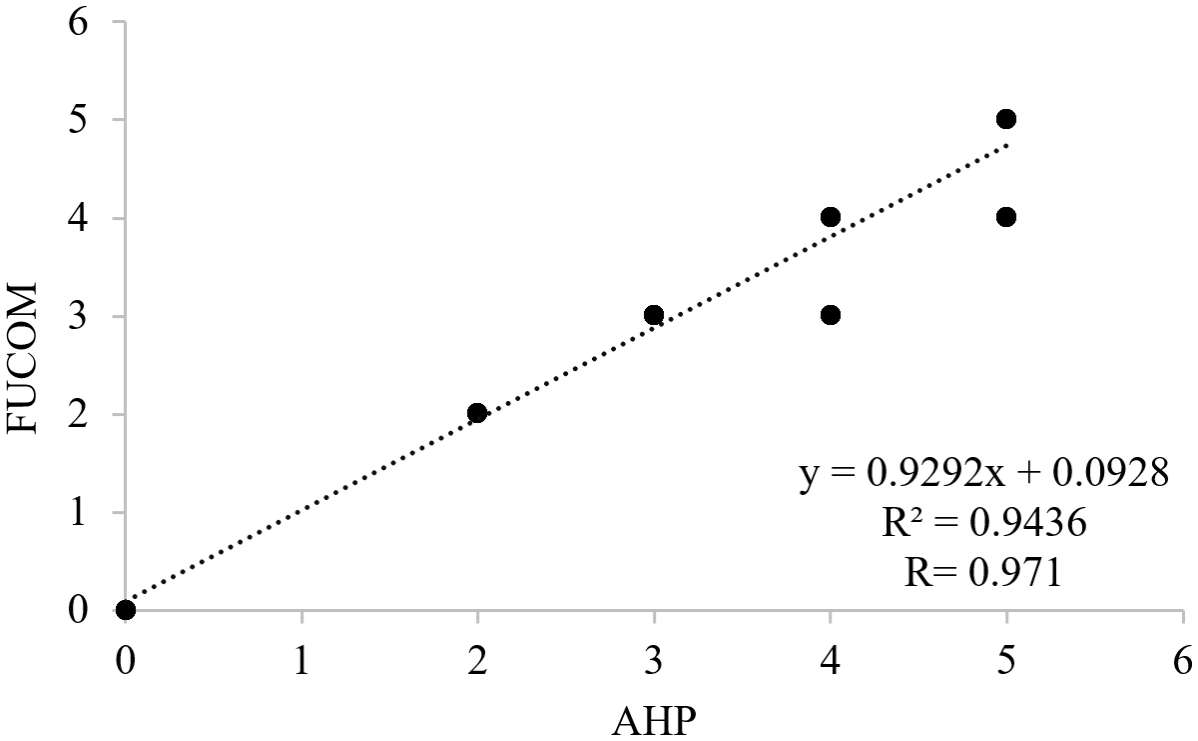
Correlation graphic of FUCOM and AHP methods. This figure illustrates the correlation between FUCOM- and AHP-derived suitability results.

AHP required 36 pairwise comparisons for the nine criteria, whereas FUCOM required only eight. This reduction demonstrates FUCOM’s efficiency in minimizing expert workload and potential inconsistency issues. Despite its simplified structure, FUCOM produced results highly consistent with AHP: the correlation of pixel values at hive locations was r = 0.971, and the standard deviation between weights was < 0.018 (Table 5 and Fig 7). Both methods therefore provided statistically robust and mutually reinforcing outcomes.

AHP remains the most widely used MCDM method in land-use studies, including apicultural suitability assessments. Its hierarchical structure allows complex problems to be decomposed systematically, while the Consistency Ratio (CR) ensures logical coherence in expert judgments. However, the large number of pairwise comparisons can increase expert fatigue and the likelihood of inconsistency, particularly when many criteria need to be evaluated.

By contrast, FUCOM offers a streamlined alternative by requiring fewer comparisons and ensuring mathematical consistency through the Deviation from Full Consistency (DFC) index. This efficiency makes it especially useful in contexts where expert time is limited. Previous applications in domains such as groundwater mapping, wind farm siting, and agricultural land evaluation [25,56–65] reported similarly strong correlations with AHP and other established methods. Nevertheless, FUCOM in its classical form has notable limitations: unlike AHP, it cannot natively accommodate hierarchical structures or explicitly model uncertainty, which may restrict its use in more complex or multi-layered decision problems.

In the present study, these trade-offs were directly reflected: FUCOM’s simplicity facilitated consensus-based evaluation with a relatively small panel of local experts, while AHP’s CR check provided an additional safeguard against inconsistency. The combined application of both methods therefore enhanced robustness, while also demonstrating that FUCOM can serve as a valid and efficient alternative to AHP in apicultural land-use planning.

### 5.3 Validation of the study and comparison with literature

Validation against real-world beekeeping records is essential for confirming the reliability of spatial suitability models [79]. In this study, 615 georeferenced hive locations were integrated into the suitability maps, with 83% falling within areas classified as moderately, highly, or very highly suitable. This high degree of overlap demonstrates the robustness of the methodology and aligns with previous findings. For example, Sarı [42] reported overlap rates of 82% for AHP, 88% for VIKOR, and 91% for TOPSIS in the Konya region, highlighting the reliability of GIS–MCDM frameworks in apicultural suitability assessments.

Statistical validation using ROC–AUC further confirmed these results. The AHP-based map achieved an AUC of 0.774, while FUCOM-based map achieved 0.754—both exceeding the 0.5 threshold for acceptable performance. These values are comparable to those reported in groundwater potential mapping (0.761–0.765) [80], although slightly lower than crop-specific studies such as citrus (0.87) [81] and pistachio (0.766) [82]. Overall, these outcomes indicate that although AHP performed slightly better, both methods yielded reliable and transferable predictions.

Finally, feedback from local beekeepers and experts provided an additional layer of validation. Their confirmation that the most suitable areas identified by the models corresponded with their field knowledge—particularly in upland districts—confirms that the generated maps are both statistically sound and practically applicable.

### 5.4 Limitations of the study and recommendations

Despite the comprehensive methodological framework adopted in this study, several limitations should be acknowledged to contextualize the findings and guide future research.

First, land use/cover emerged as the most influential factor, yet the study could not incorporate direct data on pollen and nectar availability, which are essential for sustaining bee nutrition and colony productivity. Likewise, detailed spatiotemporal records of pesticide applications were unavailable. While lowland areas were excluded to reduce pesticide-related risks, integrating explicit data on floral resources and pesticide use would improve the ecological validity of future models.

Second, the criteria and weights applied here were tailored to the ecological and socio-economic conditions of Mardin Province. Although they align with factors employed in other studies, their direct transferability is limited. Applying and recalibrating the methodology in different ecological settings would help test its adaptability and strengthen its generalizability.

Third, this study employed AHP and FUCOM in their classical (crisp) forms. While both methods produced robust and consistent results, the absence of fuzzy-based extensions limited the explicit handling of uncertainty in expert judgments. Future research could integrate fuzzy or hybrid MCDM approaches—such as Fuzzy AHP or Fuzzy FUCOM—to better capture ambiguity and enhance methodological robustness.

Finally, the validation relied on 615 hive locations collected at a single point in time. Although this provided strong empirical support, seasonal and interannual variations in resource availability and management practices may influence suitability patterns. Long-term monitoring across multiple years and seasons would strengthen the reliability of validation outcomes.

In summary, while these limitations warrant caution, they also point to clear directions for future research. Incorporating dynamic floral and pesticide datasets, testing fuzzy or hybrid MCDM methods, and applying the framework across diverse ecological contexts would broaden its applicability. Importantly, FUCOM’s efficiency suggests that it can serve as a valuable alternative in data- or time-limited settings. Continued testing alongside established methods such as AHP and newer approaches like OPARA will advance methodological diversity and strengthen decision-support frameworks for apicultural land-use planning.

## 6. Conclusion

This study demonstrated the effectiveness of integrating Geographic Information Systems (GIS) with Multi-Criteria Decision-Making (MCDM) techniques to identify optimal beekeeping areas in Mardin Province, Türkiye. By applying both the Analytical Hierarchy Process (AHP) and the Full Consistency Method (FUCOM), suitability maps were developed that incorporated environmental, climatic, topographical, logistic, and socio-economic factors. The results highlighted land use/cover, proximity to water sources, and precipitation as the most influential criteria shaping beekeeping suitability.

Validation using field-recorded hive locations and ROC–AUC analysis confirmed the reliability of the framework. A total of 83% of hive locations overlapped with areas classified as moderately suitable or higher, while AUC values of 0.774 (AHP) and 0.754 (FUCOM) indicated strong predictive performance. Although AHP showed slightly higher accuracy, FUCOM produced comparable results with far fewer comparisons, highlighting its efficiency in contexts where expert time and resources are limited.

Overall, the findings provide actionable insights for policymakers, decision-makers, and local beekeepers by identifying priority zones for sustainable apicultural practices. Beyond its regional relevance, the methodological framework can be adapted to other ecological contexts worldwide, supporting sustainable land management and pollinator conservation. Future research should integrate dynamic datasets on floral resources, pesticide applications, and seasonal variability, and explore fuzzy or hybrid MCDM approaches to further strengthen methodological robustness and adaptability.

## Supporting information

S1 Fig Field photographs of beekeeping activities in Mardin Province. Images illustrate five selected hive locations recorded with GPS during field surveys. In total, 615 hive locations were visited across the province, and these photos represent a small subset of the surveyed sites. All photographs were taken by the authors during fieldwork conducted for this study. The images are original, have not been previously published, and contain no copyrighted or proprietary material. Written consent for publication was obtained from all recognizable individuals, and the authors hold full copyright for these images under the CC BY 4.0 license.
(TIF)

S1 Table Suitability class areas (hectares) per district using the AHP method. This table summarizes the spatial distribution of suitability classes across districts based on AHP-derived weights.
(XLSX)

S2 Table Suitability class areas (hectares) per district using the FUCOM method. This table presents the district-level distribution of suitability classes calculated using FUCOM-derived weights.
(XLSX)
